# Zika Virus after the Public Health Emergency of International Concern Period, Brazil

**DOI:** 10.3201/eid2804.211949

**Published:** 2022-04

**Authors:** Laith Yakob

**Affiliations:** London School of Hygiene and Tropical Medicine, London, England

**Keywords:** Zika virus, viruses, vector-borne infections, Brazil, epidemiology, arboviruses, statistics

## Abstract

More than 100,000 Zika virus cases have been reported in Brazil since the Public Health Emergency of International Concern period ended in 2016. We analyzed cases in Brazil during 2017–2021 to identify transmission trends and forecast future infection hotspots. Our results can be used for targeted interventions to reduce transmission.

Zika virus (ZIKV) is a flavivirus transmitted through the bite of mosquitoes, principally *Aedes aegypti*. The World Health Organization (WHO) declared a Public Health Emergency of International Concern (PHEIC) on February 1, 2016, by which time autochthonous ZIKV transmission had been reported in 22 countries and territories in Latin America and the Caribbean (*1*). The PHEIC was prompted by the reporting 2 months earlier of a suspected link between ZIKV infection during pregnancy and subsequent birth defects, most notably microcephaly (*2*). A high proportion of asymptomatic or mild infections (*3*) coupled with diagnostic test cross-reactivity (*4*) obscured the true number of cases during the outbreak. Accounting for these sources of uncertainty recent modeling suggests 132.3 million (95% CI 111.3–170.2 million) persons in the Americas had been infected by the end of 2018 (*5*).

WHO terminated the emergency in November 2016. During the next 5 years, 2017–2021, infection rates decreased substantially; for example, the United States reported 15 new local cases in 2017 and none since (*6*). However, ZIKV did not disappear from the region; >150,000 cases were reported, unadjusted for underreporting, in the Americas through September 2021 (*7*). The persistence of ZIKV in this region coupled with its explosive epidemic potential has meant ZIKV remains in the WHO list of priority diseases for research and development in emergency contexts (*8*). We explored post-PHEIC ZIKV transmission in Brazil, the country with the highest number of infections during the epidemic in the Americas. Our first objective was to analyze post-PHEIC infection trends in Brazil; the second was to identify consistent places and times for ongoing transmission for intervention targeting.

## The Study

**Figure 1:**
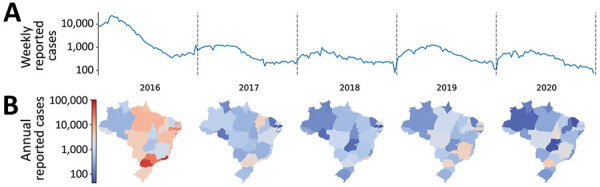
Weekly reported Zika cases in Brazil, 2016–2020. A) Log scale of reported cases during and after the Public Health Emergency of International Concern period, which ended in November 2016. B) State-level distribution of cases for each year, as reported to the Sistema de Informação de Agravos de Notificação (*9*) database (bottom).

All data were anonymized and reported at the municipality level. We obtained ZIKV infection data from the online database of the Brazilian Ministry of Health’s notifiable diseases information system (Sistema de Informação de Agravos de Notificação, SINAN) (*9*). We used data from laboratory-confirmed (either by real-time PCR or IgM serology) and clinically diagnosed cases. (Figure 1).

We adapted an autoregressive integrated moving average model to enable direct modeling of the seasonal component of time series data. We tested our seasonal autoregressive integrated moving average (SARIMA) model on monthly cases from January 2017–December 2019 and then validated it with 2020 data before using it for forecasting for 2021–2022. Parameterization involved fitting data to alternative SARIMA models by a modified Powell method and selecting the model with lowest Akaike Information Criterion. We assumed a seasonality lag of 12 time steps (i.e., seasonality repeats every 12 months) for SARIMA models and trained and validated the model for each of the 5 regions of Brazil.

Diagnostic plots for the SARIMA models comprise the standardized residuals, normal Q-Q plot, and the correlogram; all generally indicated good model fits for the data from each region (Appendix Figures 1–5). For 2021–2022, we forecasted the following case numbers for each region: North, 291 (95% CI 0–5,334); Northeast, 5,933 (95% CI 251–17,009); Southeast, 1,228 (95% CI 0–21,793); South, 291 (95% CI 28–208); and Central-West, 733 (95% CI 0–10,388) (Appendix Figure 6). We recorded final model specifications and the root mean squared errors for the 2020 SARIMA predictions (Appendix).

**Figure 2:**
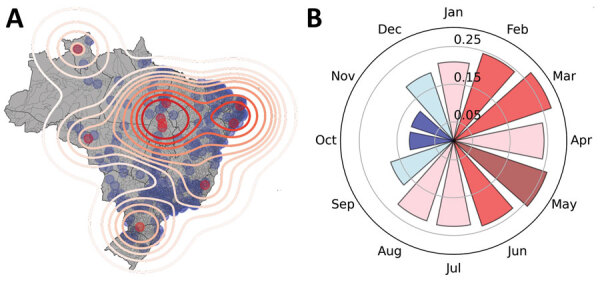
Geographic and temporal distribution of reported Zika cases after the Public Health Emergency of International Concern period, Brazil, 2017–2020. A) Consistency-weighted kernel density estimates. Contours generated with bandwidth determined by Scott’s rule adjusted by a factor of 0.7 (*12*), overlaid on the distribution of all case data. Points show centroids of municipalities that reported infections to the Sistema de Informação de Agravos de Notificação (*9*) database. B) Monthly Moran *I* statistic, estimated at the state level. Red indicates p<0.05; blue indicates p>0.05.

We applied a data filter to 5,570 municipalities in Brazil, and included in our analysis those that have reported ZIKV infections consistently every year since 2017. We generated a kernel density plot (*10*) for these areas of consistent transmission and weighted the data by minimum annual rate of infection, standardized to local municipality population; the latest census was >10 years ago, so we used 2020 estimates produced by the Brazilian Institute of Geography and Statistics (*11*). We identified centroid locations of all municipalities that reported ZIKV cases after 2016 and the hotspots of sustained transmission during the post-PHEIC period, in which >40 cases were reported per 100,000 population consistently each year (Figure 2, panel A). We generated contours from kernel density estimates weighted by the minimum annual rate of infection standardized to local municipality population. We noted hotspots in Roraima and Tocantins (North region), Alagoas (Northeast), Rio de Janeiro (Southeast), Paraná (South) and the border between Rondônia (Northern) and Mato Grosso (Central-West).

We calculated the Moran *I* statistic (*13*) for infections at the state level to determine significant disease clustering; to do so, we used a permutation approach because of reduced sensitivity to potential violations of the analytical version’s assumptions, making it a more robust metric. We calculated a reference distribution for the statistic under the null hypothesis of spatial randomness by randomly permuting the observed values over the locations. We computed Moran *I* for each randomly shuffled dataset; from the statistic, we plotted a reference distribution and a pseudo p value. Because of the high number of municipalities in Brazil with missing data, we estimated global clustering at the state level. Distrito Federal is contained entirely within the state of Goiás, and so data on its cases and population were merged with Goiás. We estimated a Moran *I* of 0.23; Monte Carlo simulation generated pseudo p value was 0.047, providing support for clustering at this level (Appendix Figure 7). We then assessed clustering for each calendar month (e.g., we included cases from January 2017, January 2018, January 2019, and January 2020 as Jan) (Figure 2, panel B). Monthly Moran *I* showed clustering persisting throughout much of the year (January–August) but breaking down when case numbers naturally waned during the low mosquito season (September–December).

## Conclusions

The objectives of this study were to identify current trends in ZIKV transmission in Brazil and to identify where, and when throughout the year, ZIKV is most consistently reported in the post-PHEIC period. Limitations of this analysis include the high rates of underreporting known to occur for ZIKV infections which, coupled with difficulties in differential diagnosis, make reliable estimates for case numbers a considerable challenge (*14*). Because of these uncertainties, the forecasts in our analysis should not be viewed as prescriptive predictions of the true numbers of ZIKV infections in Brazil 2021–2022. Instead, forecasts are estimates for cases that will be reported to the notifiable diseases information system; these forecasts have the potential to accelerate public health decisions informed by the information system.

After validating the SARIMA models fitted to monthly regional notification data from Brazil, we forecasted 8,476 new cases for 2021–2022 (95% CI 279–54,732 new cases). Although the Northeast and Southeast regions were likely to continue to have the highest total infection numbers, our consistency-weighted, population-standardized rates highlighted hotspot states within all 5 regions. In the national health system, strategic programs are coordinated and specialized services are delivered at the state level; our results can help inform specific municipalities within hotspot states that exhibit consistently high-level notifications. Identifying hotspots has 2 purposes: first, it provides targets for more intensive vector control efforts to ameliorate disease burden among the worst-affected populations; second, it helps to inform site selection for seroprevalence studies and intervention trials (*15*). Temporality of clustering statistics indicating clear and consistent transmission seasonality also contributes to both of these purposes.

Appendix Additional information about Zika virus in Brazil after the Public Health Emergency of International Concern period.
