# Supplementary material for: Zika Virus after the Public Health Emergency of International Concern Period, Brazil
Source: Emerg Infect Dis. 2022 Apr;28(4):837–40. doi: 10.3201/eid2804.211949 (PMC8962915; doi:10.3201/eid2804.211949)
Supplement: Appendix — Additional information about Zika virus in Brazil after the Public Health Emergency of International Concern period. [file 21-1949-Techapp-s1.pdf]

Article DOI: https://doi.org/10.3201/eid2804.211949

Zika Virus after the Public Health Emergency of International Concern Period, Brazil

Appendix

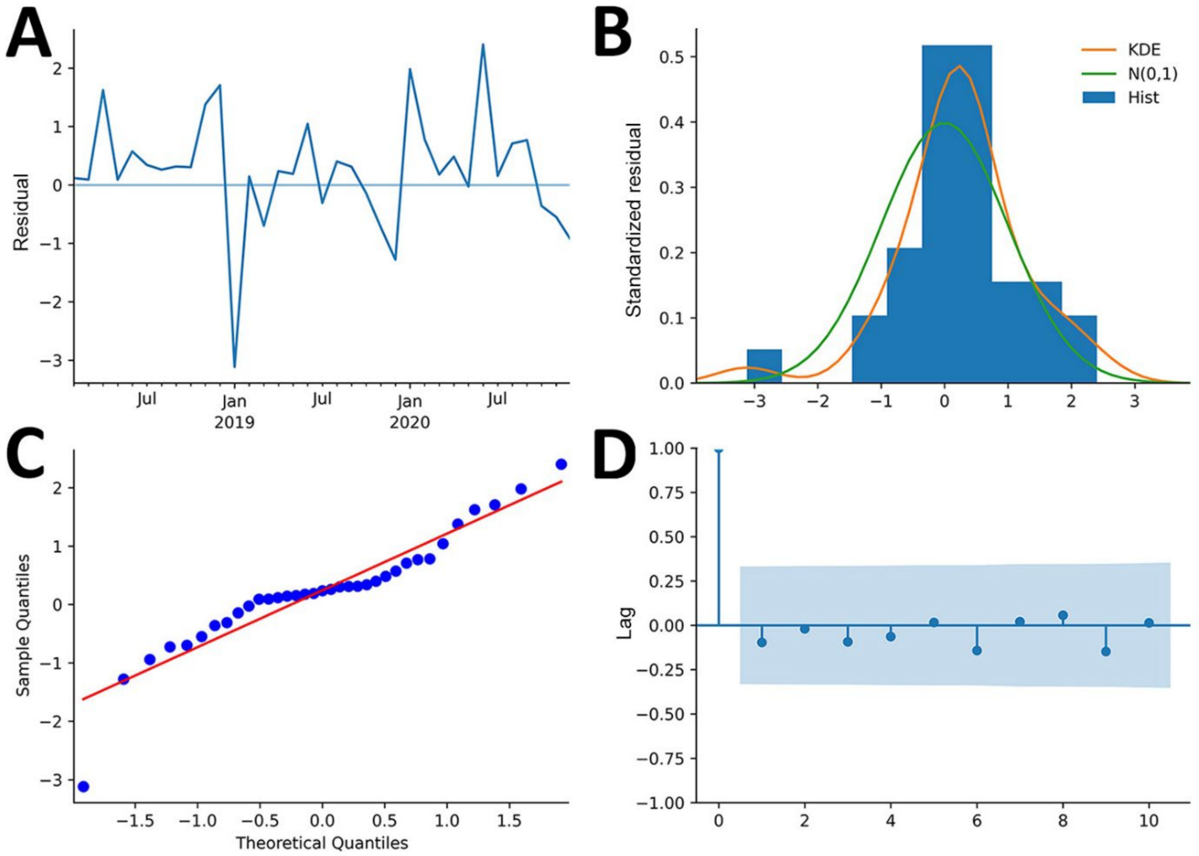

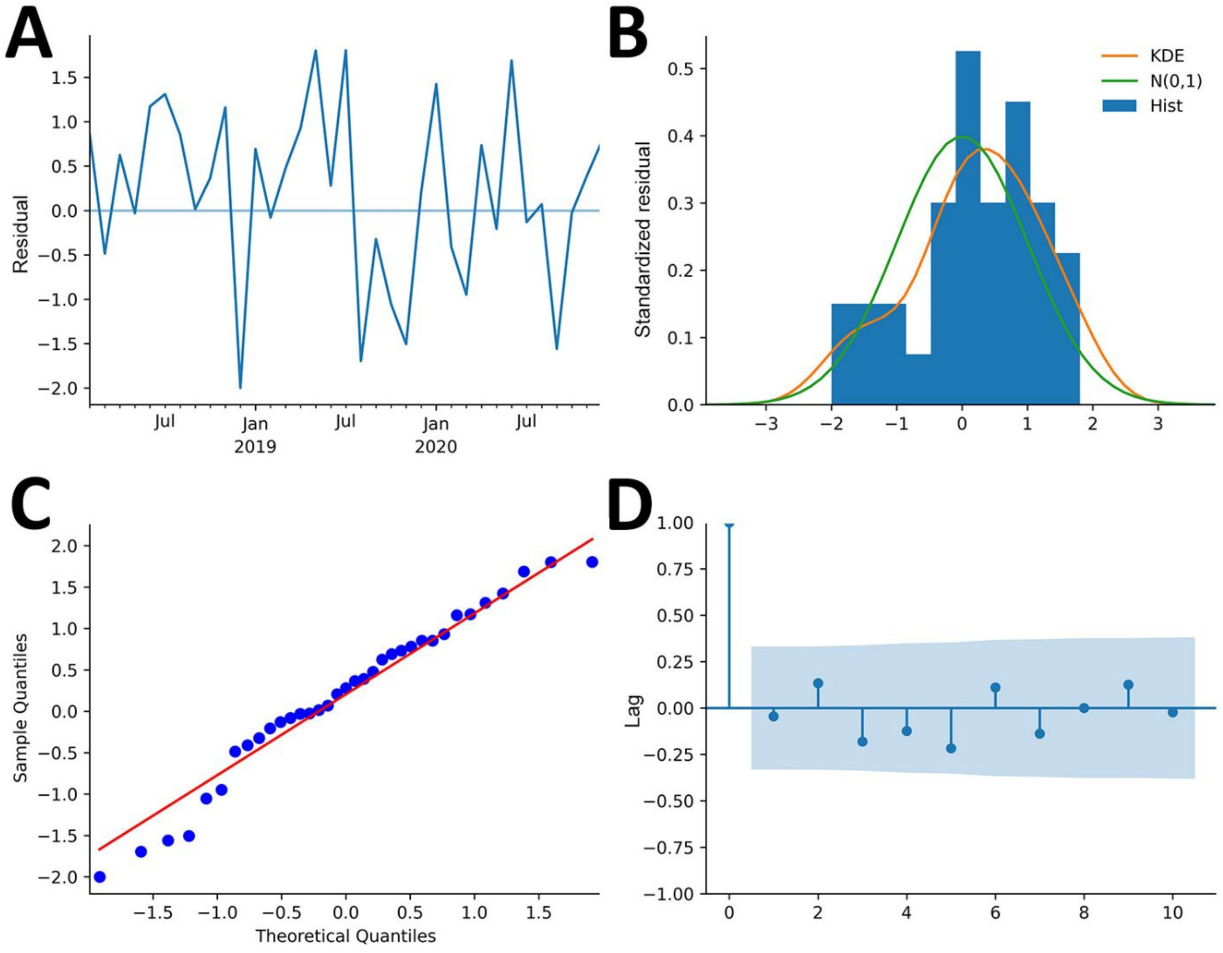
A
B
C
D
Residual
Jul
Jan
2019
2020
KDE
N(0,1)
Hist
Standardized residual
Sample Quantiles
Theoretical Quantiles
Lag

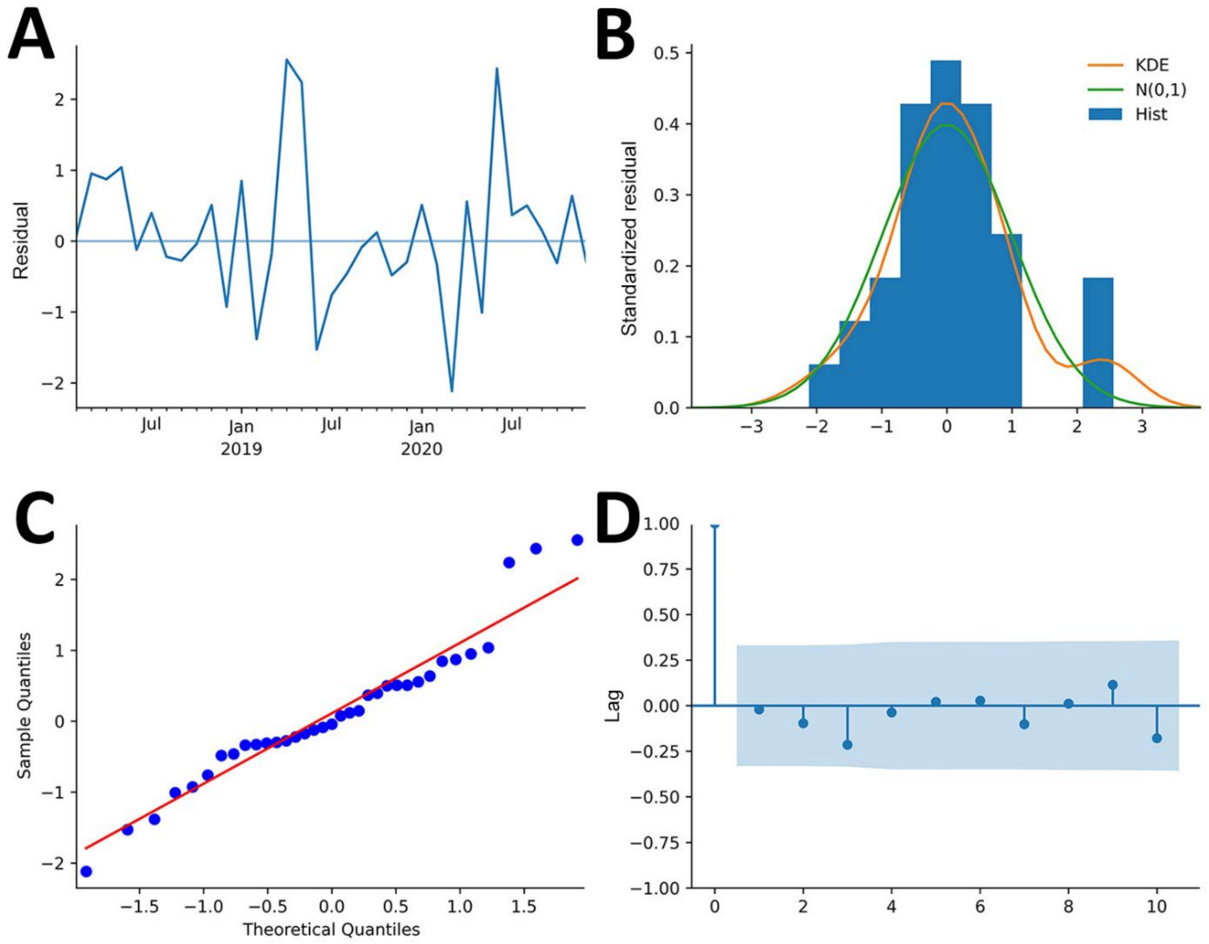
A
Residual
Jul
Jan
2019
Jul
Jan
2020
Jul
B
Standardized residual
KDE
N(0,1)
Hist
C
Sample Quantiles
Theoretical Quantiles
D
Lag

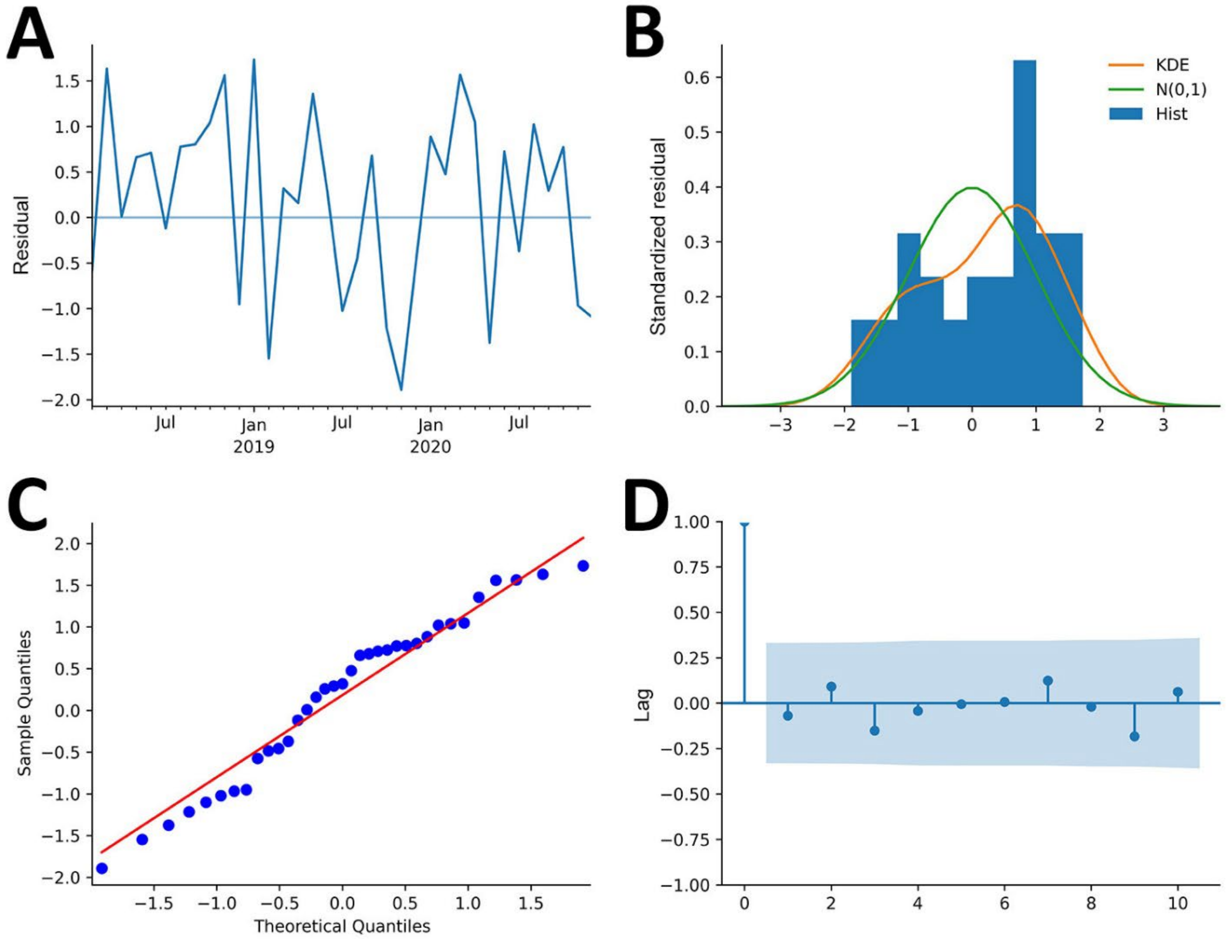
A
B
C
D
Residual
Jul
Jan
2019
2020
KDE
N(0,1)
Hist
Standardized residual
Sample Quantiles
Theoretical Quantiles
Lag

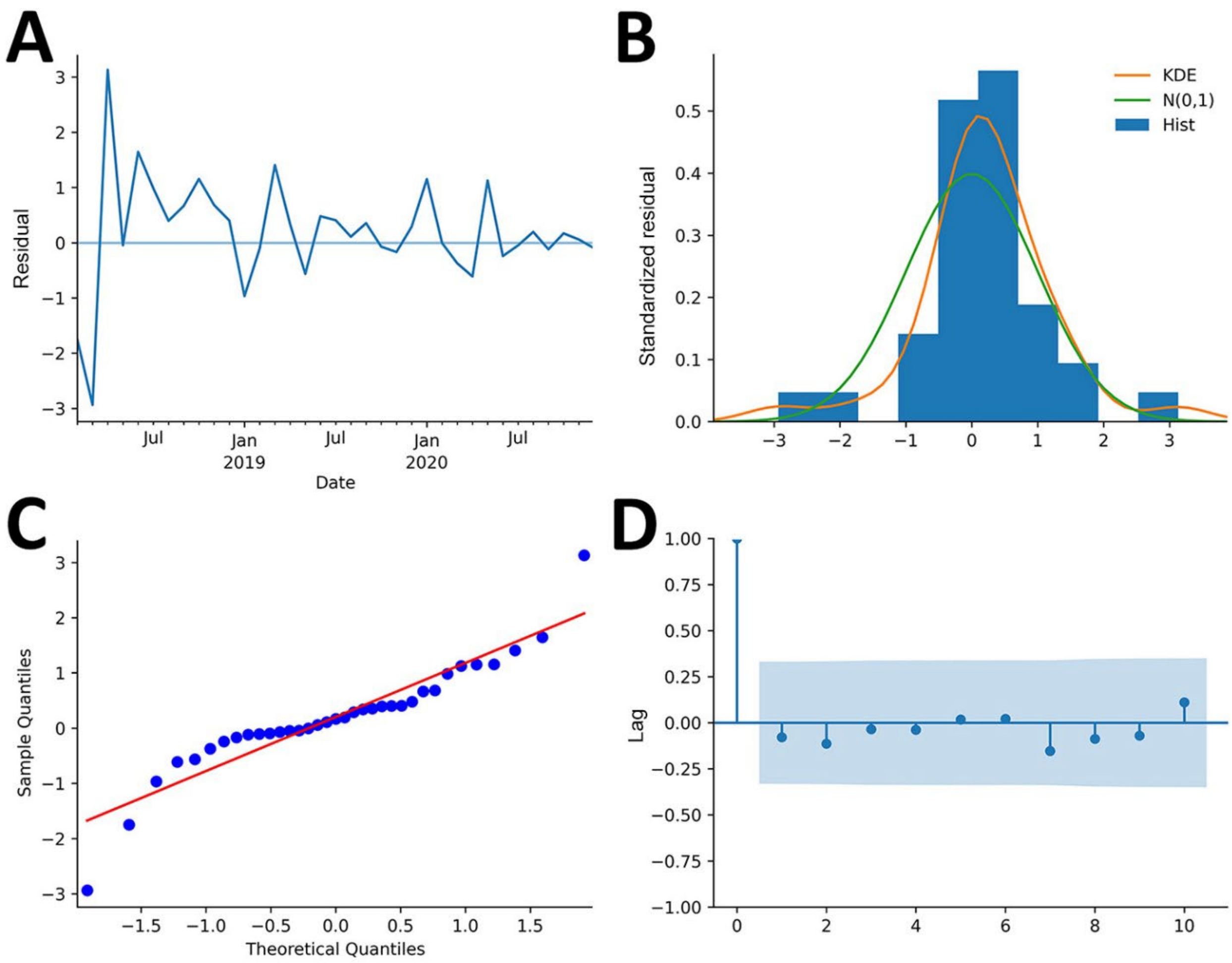
A
B
C
D
Residual
Date
Jul
Jan
2019
2020
KDE
N(0,1)
Hist
Standardized residual
Sample Quantiles
Theoretical Quantiles
Lag

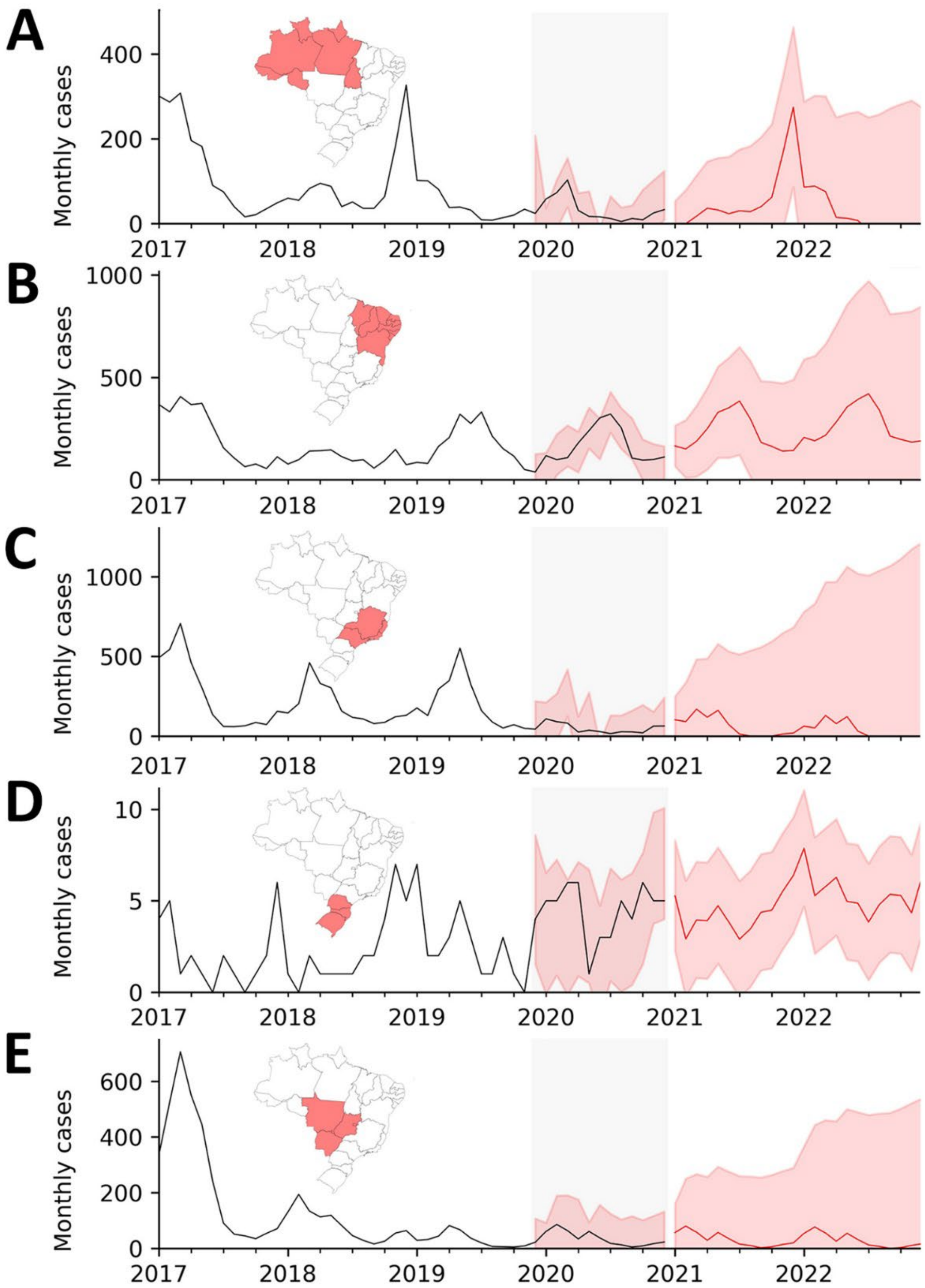

Appendix Figure 6. Seasonal autoregressive integrated moving average (SARIMA) models for Brazil's 5 regions: North (A), Northeast (B), Southeast (C), South (D), Central-West (E). Models were trained with SINAN data up until end of 2019, then validated before forecasting for 2021 and 2022. Gray shading indicates validation period. Black line indicates mean in validation period; red line indicates forecasting mean. Red shading indicates confidence bounds. Diagnostic plots show good SARIMA model fits.

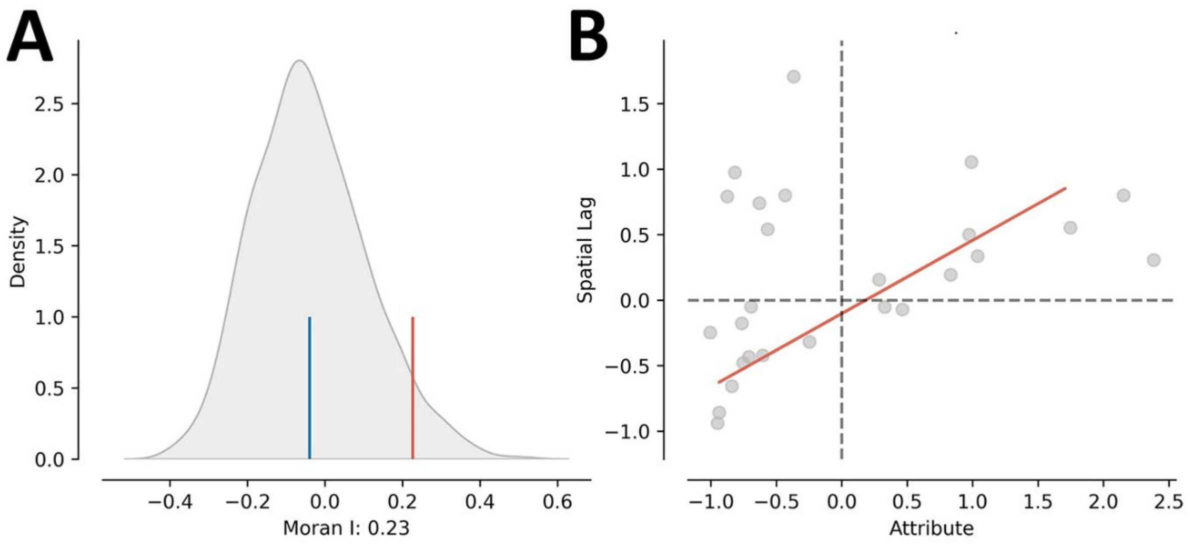
A
2.5
2.0
1.5
1.0
0.5
0.0
Density
−0.4
−0.2
0.0
0.2
0.4
0.6
Moran I: 0.23
B
1.5
1.0
0.5
0.0
−0.5
−1.0
Spatial Lag
−1.0
−0.5
0.0
0.5
1.0
1.5
2.0
2.5
Attribute
